# Effects of a Primary Care-Based Intervention on the Identification and Management of Patients with Coronary Heart Disease and Mental or Cognitive Comorbidity—A Study Protocol

**DOI:** 10.3390/ijerph20105814

**Published:** 2023-05-13

**Authors:** Lena Sannemann, Lisa Bach, Kira Isabel Hower, Peter Ihle, Kai Keller, Charlotte Leikert, Christin Leminski, Adriana Meixner, Ingo Meyer, Laura Nordmeyer, Samia Peltzer, Sophie Peter, Belinda Werner, Ludwig Kuntz, Holger Pfaff, Frank Schulz-Nieswandt, Christian Albus, Frank Jessen, on behalf of the CoRe-Net Study Group

**Affiliations:** 1 Department of Psychiatry and Psychotherapy, Faculty of Medicine and Cologne University Hospital, University of Cologne, Kerpener Str. 62, 50924 Cologne, Germany; frank.jessen@uk-koeln.de; 2 Institute for Medical Sociology, Health Services Research and Rehabilitation Science, Faculty of Human Sciences, Faculty of Medicine and University Hospital Cologne, University of Cologne, Eupener Str. 129, 50933 Cologne, Germanyholger.pfaff@uk-koeln.de (H.P.); 3 PMV Research Group, Faculty of Medicine and Cologne University Hospital, University of Cologne, Herderstraße 52, 50931 Cologne, Germany; 4 Department of Psychosomatics and Psychotherapy, Faculty of Medicine and Cologne University Hospital, University of Cologne, Weyertal 76, 50931 Cologne, Germanychristian.albus@uk-koeln.de (C.A.); 5 Department of Business Administration and Health Care Management, Faculty of Management, Economics and Social Sciences, University of Cologne, Albertus-Magnus-Platz, 50923 Cologne, Germanykuntz@wiso.uni-koeln.de (L.K.); 6 Chair of General Practice II and Patient-Centredness in Primary Care, Institute of General Practice and Primary Care, Faculty of Health, Witten/Herdecke University, Alfred-Herrhausen-Str. 50, 58455 Witten, Germany; 7 Institute of Sociology and Social Psychology (ISS), Faculty of Management, Economics and Social Sciences, University of Cologne, Albertus-Magnus-Platz, 50923 Cologne, Germanyschulz-nieswandt@wiso.uni-koeln.de (F.S.-N.); 8 Centre for Health Services Research Cologne (ZVFK), University of Cologne, Eupener Str. 129, 50933 Cologne, Germany; 9 German Center for Neurodegenerative Diseases (DZNE), Venusberg-Campus 1/99, 53127 Bonn, Germany; 10 Excellence Cluster on Cellular Stress Responses in Aging-Associated Diseases (CECAD), University of Cologne, Joseph-Stelzmann-Str. 26, 50931 Cologne, Germany

**Keywords:** heart disease, mental disorders, comorbidity, cognitive impairment, primary care, depression, anxiety, organisational characteristics

## Abstract

Mental and cognitive disorders (MCD) negatively affect the incidence and prognosis of coronary heart disease (CHD). Medical guidelines recommend the appropriate management of comorbid MCD in patients with CHD, yet there is evidence that the implementation in primary care is not always adequate. We present the protocol for a pilot study that aims to develop a minimally invasive intervention and evaluate its feasibility in the primary care setting to improve the identification and management of comorbid MCD in patients with CHD. The study consists of two consecutive parts and will be carried out in Cologne, Germany. Part 1 comprises the development and tailoring of the intervention, which is guided by qualitative interviews with primary care physicians (PCPs, *n* = 10), patients with CHD and MCD and patient representatives (*n* = 10). Part II focuses on the implementation and evaluation of the intervention in *n* = 10 PCP offices. Changes in PCP behaviour will be analysed by comparing routine data in the practice management system six months before and six months after study participation. In addition, we will explore the influence of organisational characteristics and perform a socio-economic impact assessment. The outcomes of this mixed-method study will inform the feasibility of a PCP-based intervention to improve quality of care in patients with CHD and comorbid MCD.

## 1. Introduction

Coronary heart disease (CHD) ranks among the highest drivers of global disease burden among all age groups, but specifically in the age group of 50 years and older [1]. To this date, it is considered the leading cause of mortality in Germany [2]. Mental and cognitive disorders (MCD) are among the top causes for global disease burden [1], and they also negatively affect the incidence and prognosis of CHD. The prevalence of depression is 15–30% among patients with CHD and therefore higher compared to the general public [3]. There is strong evidence that depression and anxiety are risk factors for negative disease prognosis and mortality [3,4,5], and they negatively impact quality of life [6] and reduce adherence to necessary lifestyle changes and treatment [7]. In addition, interactions between cognitive impairment or dementia and CHD have been observed. On the one hand, cognitive deficits are considered risk factors for non-adherence to therapy [8] and may therefore impair disease prognosis, while on the other hand, CHD has been shown to increase the risk of subsequent dementia [9]. Current guidelines therefore recommend appropriate diagnosis and treatment of comorbid MCD in patients with CHD [4,7,8,10].

The identification and management of both mental and cognitive disorders are common responsibilities of primary care physicians (PCP). However, there is evidence that guideline recommendations are not adequately implemented in primary care. A meta-analysis on the ability of PCPs to diagnose depression indicated that PCPs showed a diagnostic sensitivity of 47.3–50.1%, while the rate of correctly identified non-depressed individuals was higher (81.3%) [11]. This is in line with data from the “Quality of health care with regard to detection and treatment of mental disorders in patients with coronary heart disease” (MenDis-CHD) study [12]. That study showed that, out of 102 patients who were diagnosed with a mental disorder by the study team, 51% had not yet received a diagnosis from their physician [13]. Several causes for underdiagnoses of dementia in the primary care setting have been identified, including lack of adequate training, fear of stigma, and time and resource constraints [14,15,16]. However, while identification of symptoms is important and the premise for subsequent actions, it needs to be followed up by adequate management and treatment procedures. Moreover, quality of care strongly relies on how MCDs are addressed within the patient–carer relationship.

For this pilot study, we aim to develop and test the feasibility of an intervention to improve the identification and management of MCD in patients with CHD in the primary care setting. The rationale is largely based on results from the cross-sectional MenDis-CHD study [12,13,17]. It is part of the interdisciplinary Cologne Research and Development Network (CoRe-Net), a competence network of practice and research for the model region of Cologne [18]. The network is funded by the German Federal Ministry of Education and Research (BMBF) and based on the value-based health care approach [19,20,21].

## 2. Materials and Methods

### 2.1. Study Setting

This study is part of the CoRe-Net research projects MenDis-CHD II, a study on quality of care in diagnosis and therapy of MCD in CHD, and OrgValue II, a study on characteristics of value-based care from the perspective of care institutions. It consists of two consecutive parts. Part I aims to develop a minimally invasive intervention to improve identification and management of MCD in CHD patients in the primary care setting. Additionally, potential determinants of implementation in PCP offices will be identified. In this context, “minimally invasive” refers to an intervention that hardly interferes with routine care practice and is designed to be time- and cost-efficient. The intervention will then be implemented and evaluated in PCP offices in Cologne, Germany and surrounding suburban municipalities in part II of the study. The total duration of Part II is twelve months, divided into a six-month pre-intervention observation period and a six-month intervention period. Our aims are to evaluate the feasibility of the intervention and, if given, assess whether the intervention changes PCPs’ management of CHD patients.

#### 2.1.1. Part I: Intervention Development and Tailoring

**Participants.** Interviews with PCPs in Cologne (*n* = 10), patients with CHD and MCD and patient representatives (i.e., representatives of patient organisations and self-help groups, *n* = 10) will be conducted to tailor and adapt the minimally invasive intervention and develop an implementation programme. It is assumed that information saturation is reached at about 10 interviews per group.

Inclusion criteria:

PCPs:

- Office located in Cologne or surrounding suburban municipalities

Patients:

- Diagnosis of CHD and comorbid MCD disease
- Proficient in the German language
- ≥18 years old
- Able to provide written informed consent

Patient representatives:

- Proficient in the German language
- ≥18 years old
- Able to provide written informed consent

**Recruitment.** PCPs will be recruited via multiple channels. We will contact participants of PCP symposia organised by the University Hospital Cologne who consented to be contacted via e-mail. In addition, PCPs in Cologne and surrounding suburban municipalities will be contacted by phone via their publicly accessible contact information. Patient recruitment will be based on the MenDis-CHD I patient sample. Patient representatives will be recruited via the CoRe-Net network.

**Intervention development.** The overall aim of the intervention is to enhance awareness of mental and cognitive symptoms related to CHD in PCPs and patients and thereby stimulate improved diagnosis and management. The intervention will be composed of different elements: A trigger question, screening tests, information material for patients and PCPs and a training course for PCPs. The design of the intervention elements will be tailored to the PCPs’ needs and preferences and take into consideration the limited time resources available in PCP offices.

**Figure 1:**
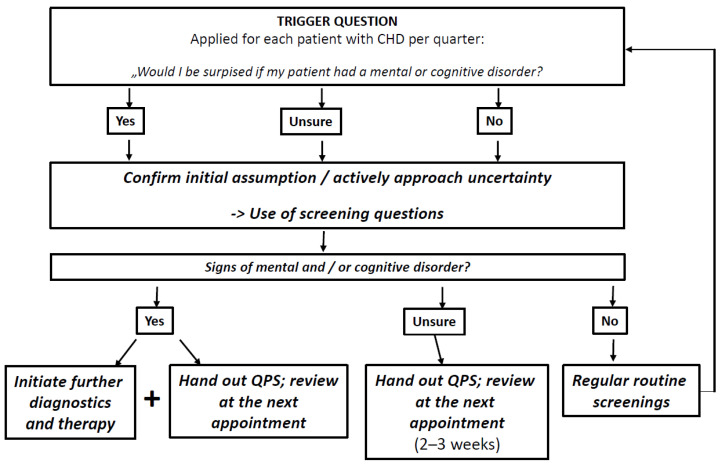
Screening flow chart for PCPs to guide their actions after the use of a trigger question. CHD Coronary heart disease, QPS Question prompt sheet.

Trigger question: The trigger question will be adapted from the 12-month Surprise Question (SQ-12) known from palliative care research (“Would I be surprised if this patient died in the next 12 months?”) [22,23,24,25,26,27,28]. The SQ-12 has originally been designed to identify individuals eligible for palliative care and prompt treatment decisions. It will be adapted to fit our research question (“Would I be surprised if my patient had a mental or cognitive disorder?”). Based on previous experiences with the SQ-12, we assume that the focus of the question on surprise will stimulate reflection in the decision-making process and challenge routinised assumptions and patterns of behaviour. Therefore, PCPs should ask themselves the trigger question for each patient with CHD. As a result, we expect it to trigger consecutive actions as described in Figure 1, which are in line with recent diagnostic guideline recommendations for the identification and management of MCD [8,10,29,30,31]. PCPs should either confirm their assumption of the presence of MCD or actively approach uncertainty by using screening methods and following up with further diagnostics, therapeutic actions and information for the patients, depending on the screening result.

Booklet and screening material: In addition to the trigger question, a booklet will be designed for the PCPs participating in the study. It will include detailed information on the latest guideline recommendations regarding the detection of and care for MCD in patients with CHD and further assistance in dealing with the intervention. Additionally, PCPs will be provided with screening tools for time-efficient identification of potential cognitive difficulties and depressive or anxiety symptoms. Screening for depression or anxiety will be initiated by an open question on how patients are currently feeling, followed by the PHQ-4 as a brief screening scale with two items on depression and two items on anxiety [32], and another open question on potential further mental health problems. Screening for cognitive impairment will consist of a question on subjectively perceived cognitive decline and worries associated with it. In addition, the Six-Item-Screener is proposed as an easy-to-use screening tool that can be completed in about one minute and followed up by more detailed cognitive tests if necessary [33].

Training course: A training course for PCPs will be designed to increase knowledge and raise awareness for the common comorbidity of CHD and MCD. Furthermore, the training course will serve to familiarise the PCPs with the study materials. The training course will be led by senior investigators (FJ, CA) who are specialists in psychosomatic medicine and psychiatry and have longstanding expertise in CHD and PCP research. To harmonise the training course and ensure reliability, a presentation will be developed that serves as a guideline. Details of the training course, such as length, specific contents or the participation of staff, will be tailored to the results of the qualitative interviews.

Question prompt sheets: We will develop a question prompt sheet (QPS) for patients. A QPS provides adequate support for communication between patients and providers. Its use can empower patients to take up an active role in communication with their PCPs, ask questions and thus receive more information. This increases patient knowledge and has a positive effect on patient satisfaction and the physician-patient relationship [34,35]. It also reduces potential patient anxiety without increasing the consultation time [35,36,37]. The QPS will provide evidence-based information about the comorbidity of CHD and MCD, examples of relevant questions, space for personal notes and information on further resources or points of contact.

#### 2.1.2. Part II: Intervention Implementation

**Participants.** For the implementation of the minimally invasive intervention designed in Part I, we intend to enrol *n* = 10 PCP offices (single or group practices). The sample size is based on calculations of the number of patients with CHD per practice per quarter. Based on previous reports, we assume that the number of patients per quarter per PCP will be 900 [38]. Data from the DETECT study on 55,518 patients in German primary care showed that 12.4% were diagnosed with CHD [39]. As a conservative measure, we decided on an estimate of 10% CHD patients, yielding *n* = 90 CHD patients per PCP per quarter. Multiplied by the number of PCP offices (*n* = 10), we expect 900 CHD patients per quarter, providing us with 1800 cases in the six-month pre-intervention observation period and 1800 cases in the six-month intervention period. To control for the influence of gender, we aim to balance recruitment of PCPs.

Inclusion criteria:

- Single or group PCP office in Cologne or the surrounding suburban municipalities

Exclusion criteria:

- Advanced training in basic psychosomatic care
- Specialist additional qualification of psychotherapy
- Participation in an interview in Part I

**Recruitment.** Recruitment strategies will focus on several channels, including presentation of the project at PCP events or symposia, recruitment via CoRe-Net, and invitation e-mails or letters to local PCP networks and PCP offices associated with the University Hospital Cologne.

**Intervention implementation.** PCPs will be invited to a training course as developed in Part I of the study. They will be encouraged to use the study material as described above during the six-month intervention period to aid them in identifying and managing CHD patients with potential MCD. However, we will not monitor usage and will instead emphasise that the intervention should be implemented in accordance with the PCPs’ routine work structure. By doing so, we aim to stay close to a “real-world setting” to analyse feasibility and increase generalisability of the results. After completing the intervention period, each PCP will be interviewed to assess their experiences and evaluate the feasibility of the intervention.

### 2.2. Outcomes

#### 2.2.1. Primary Outcome

The primary objective of this study is to evaluate the feasibility of the intervention in the PCP setting. Using a qualitative approach, we will evaluate feasibility and acceptance as well as barriers and facilitators of the implementation. Routine care data will quantitatively inform about the number of patients with CHD potentially reached by the minimally invasive intervention. In addition, we will assess recruitment success and the rate of study completion.

#### 2.2.2. Secondary Outcomes

- Changes in PCP behaviour, operationalised by primary care routine data in the practice management system related to MCD. This includes patient-level information on diagnoses, medication prescription, referral to specialists, and billing codes of diagnostic and therapeutic measures related to MCD.
- Influence of organisational characteristics on patient-level outcomes of the implemented intervention, operationalised by a daily workload statistic (i.e., the total number of patients treated per day per PCP) derived from routine data and a questionnaire assessing basic information regarding the participating PCPs, practice structures and treatment processes.
- Cumulative socio-economic return calculated by a formative socio-economic impact assessment (SEIA) for different implementation scenarios to support future implementations strategies.

### 2.3. Data Collection

Data in Part I of the study will be collected from individual narrative interviews using a semi-structured interview guide. Interviews will take place face-to-face or via videoconference and are scheduled for approximately 60 min. Previous studies indicate that data quality from video interviews is not of inferior quality compared to traditional, face-to-face interviews and that rapport is comparable [40,41]. Two researchers who are trained in semi-structured interviewing will conduct the interviews. Interviews will be recorded, transcribed verbatim by an external professional transcription service and anonymised. The semi-structured qualitative interview guide [42] includes three thematic blocks of approximately 20 min each: (1) experiences and requirements for identification and standardised care of patients with CHD and MCD, (2) presentation/discussion of the planned intervention and (3) barriers and facilitators of planned intervention and of the implementation in PCP offices. During the interviews, participants will be presented with drafts of the booklet and the QPS for patients.

**Figure 2:**
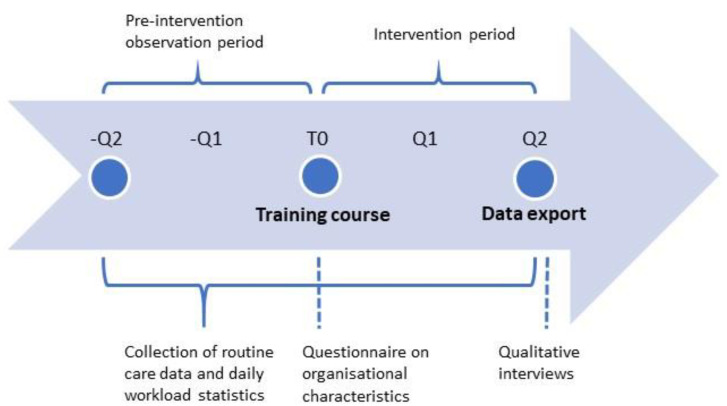
Study flow chart of Part II including time points for data collection. *Q* Quarter, *T0* study onset (training course).

For Part II, qualitative and quantitative data will be collected at different time points. A detailed description of the variables is provided in Table 1. Organisational characteristics will be collected via questionnaire at the beginning of the intervention period and entered into electronic case report forms. Anonymous routine care data as well as the daily workload statistics per PCP will be exported from practice management systems via a service provider. The data export will take place once per PCP office for each PCP separately at the end of the intervention phase and retroactively extract all relevant information from the preceding twelve months (six-month pre-intervention observation period and six-month intervention period). The day of the training course with the PCP marks the start of the six-month intervention phase. Qualitative interviews to evaluate the intervention will be conducted in line with the procedure of Part I described above. A schematic depiction of the study flow and data collection is provided in Figure 2.

### 2.4. Data Management

**Figure 3:**
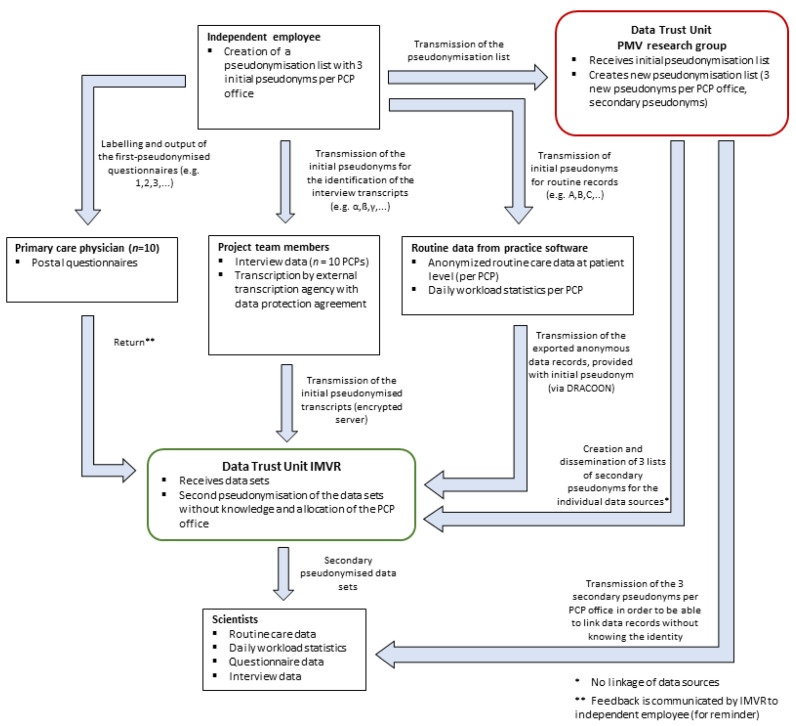
Data flow and conceptualisation of the data protection concept for all three data sources per PCP office in Part II of the study (questionnaire, interview, routine care data and workload statistic). PCP Primary care physician.

Data management is carried out in accordance with the General Data Protection Regulation (GDPR). Given that our study design includes three different data sources per PCP office (questionnaire, qualitative interview and routine care data), we developed a detailed data protection concept that allows us to connect data from all three sources without identification of the underlying practice. Two independent data trust units within CoRe-Net will be responsible for storing the data, pseudonymising it twice and providing it to the researchers for analysis. A detailed description of the data protection concept is provided in Figure 3.

### 2.5. Data Analysis

Data analysis will be based on a combination of quantitative and qualitative analysis methods. The transcripts of the interviews in Parts I and II will be entered into MAXQDA software (VERBI GmbH, Berlin, Germany) and analysed following Miles and Huberman [42]. The software program MAXQDA will be used for the qualitative data analysis and supports the systematic evaluation and interpretation of the transcript. Qualitative content analysis will be chosen to explore the unique perspectives of the participants and to systematically extract the content to a descriptive level. To provide validity of data interpretation, transcripts will be independently coded by two researchers. A coding framework will be developed that combines deductive and inductive approaches. The deductive approach will draw on extant concepts for implementation of patient-centred care [43] as well as the Consolidated Framework for Implementation Research [44].

Descriptive and explorative methods will be used to analyse the quantitative routine data. To investigate the influence of the intervention on PCP behaviour, we will conduct repeated measures analyses to compare the frequency of diagnostic and therapeutic actions, referrals and medication prescription before and after the training course. In this context, the influence of organisational characteristics as well as the intervention adherence, as reported in the interviews, will be taken into consideration. In addition, we will record and analyse reasons for dropout or non-adherence to the study procedures. Data from PCP offices will be included in the analysis if a minimum of 80% (≥20 weeks) of the intervention phase has been completed before dropout. To reduce the risk of dropout, regular check-ins with the PCP offices will be carried out via e-mail or phone.

In case of missing data in the questionnaires on organisational characteristics, PCPs will be re-contacted to fill in the missing data. To reduce the risk of incomplete data in routine care datasets, as recommended in a study design phase [45], we have carefully restricted the export to meaningful variables relevant to our research question that are consistently collected in routine clinical practice. Additionally, we have made provisions to ensure that the extraction filters also identify the relevant data in case of spelling mistakes in the documentation. Prior to analysis, plausibility checks will be carried out on routine care data exports to rule out technical errors during the export process that may lead to incomplete data.

For the socio-economic impact assessment, primary and secondary data on the effects of the intervention will be collected and evaluated according to the principle of cost-benefit analysis [46]. A methodological framework is used in conjunction with evaluation software originally developed for business model development for IT-supported utility services [47].

### 2.6. Quality Assurance and Safety Provisions

We estimate the risk of the study to be low. The study does not introduce new care or therapy methods; instead it analyses whether the intervention changes the frequency of established routine care procedures in the PCP setting. We do not expect any adverse events.

### 2.7. Ethics and Data Protection

The study has been approved by the Ethics Commission of the Faculty of Medicine of Cologne University under the ID 21-1530. It has been registered at the German Clinical Trials Register under the ID DRKS00022154. The study will be conducted in accordance with the Declaration of Helsinki, the General Data Protection Regulation (GDPR) and national data protection law. All participating PCPs will receive written study information and provide informed consent prior to participation.

## 3. Discussion

In the German health care system, PCPs are primarily responsible for providing long-term care for patients with CHD and coordinating the patients’ trajectories [8]. Therefore, it is highly relevant to raise awareness about the relationship between MCD and CHD and increase guideline adherence among physicians in the primary care setting. We assume that this goal will be achieved by the combination of the intervention components. In addition to emphasising guideline recommendations, the training course will teach PCPs about the prevalence of MCD in patients with CHD and provide evidence on their interaction regarding negative clinical outcomes. The application of the trigger question will presumably increase awareness further and challenge routinised patterns of behaviour, thereby leading to increased screenings for MCD and subsequent diagnostic and therapeutic actions. In addition, the QPS for patients aims at empowering them to ask questions about the connection between MCD and CHD and better understanding symptoms of MCD that they might experience. Consequently, this should influence the communication between patients and PCPs and ultimately contribute to improved patient-physician relationship.

One of the strengths of this study lies within the unique combination of quantitative and qualitative methods. This allows us to put PCPs’ behaviour, as operationalised by routine care data, into context with their attitudes on the intervention and experiences with barriers and facilitators as expressed during the interviews. Combined with data on organisational determinants of the implementation, we expect the study to provide us with comprehensive results that can guide the development of a subsequent RCT.

### Limitations

The study outlined is a small pilot study in a large German metropolitan city. After the evaluation of feasibility, the results will have to be validated in a large sample of PCP offices, including offices in rural areas. Furthermore, this pilot study does not have a control group, which reduces the ability to determine the effect of the intervention.

While German guidelines for CHD recommend routine screening for affective symptoms [8,10], others have criticised routine depression screenings in CHD due to a lack of evidence on the benefits on depression or cardiac outcomes [48]. The German S3 guideline on dementia currently does not recommend routine screening for cognitive deficits [30]. However, monitoring changes in cognition is necessary to rule out potentially reversible causes for cognitive decline. Furthermore, timely diagnosis of dementia is considered the basis for treatment and care [30] and enables patients and their carers to receive access to education, support and both pharmacological and non-pharmacological treatments [49,50,51]. To address these aspects, our study design does not solely focus on the identification of MCD, but also emphasises the relevance of the quality of communication between patient and physician and the importance of subsequent diagnostic and therapeutic actions. Furthermore, the concept of shared decision-making will guide the development of screening material in the intervention.

## 4. Conclusions

To our knowledge, this pilot study is the first to apply a primary care-centred intervention aimed at improved identification and management of comorbid MCD in patients with CHD. The results will provide information about the feasibility of such a minimally invasive intervention and provide data on changes in PCP’s behaviour and organisational determinants of implementation.

## Figures and Tables

**Table 1:** Overview on organisational variables and routine care data collected during Part II of the study.

Source	Category	Variable
Questionnaire on organisational characteristics	Personal information	Gender of PCP
		Job title
		Work experience
		Organisational affiliation
	Information on the PCP office	Organisational structure
		Co-operations
		Opening hours, capacity
		Number of consulting rooms
		Number of waiting seats
		Human resources
		Mean sick days/year
		Mean continued education days/year
		Frequency of team meetings
		Control statistics (free text)
		Participation in disease management program CHD
		Home visits
		Telemedicine
		Demand (mean waiting time for appointment (days))
		Proportion of patients without appointment (%)
		Mean duration of stay in the PCP office (min)
		Mean waiting time (min)
		Mean duration of treatment (min)
		Other organisational aspects (free text)
Routine care data—diagnoses	Coronary heart disease	I20.- Angina Pectoris
		I21.- Acute myocardial infarction
		I22.- Subsequent myocardial infarction
		I23.- Certain current complications following acute myocardial infarction
		I24.- Other acute ischemic heart diseases
		I25.- Chronic ischemic heart disease
	Dementia	F00.- Dementia in Alzheimer’s disease
		F01.- Vascular dementia
		F02.- Dementia in other diseases classified elsewhere
		F03.- Unspecified dementia
		F06.7 Mild neurocognitive disorder due to known physiological condition
		G30.- Alzheimer’s disease
	Mood [affective] disorders	F30.- Manic episode
		F31.- Bipolar affective disorder
		F32.- Depressive episode
		F33.- Recurrent depressive disorder
		F34.- Persistent mood [affective] disorders
		F38.- Other mood [affective] disorders
		F39.- Unspecified mood [affective] disorder
	Neurotic, stress-related and somatoform disorders	F40.- Phobic anxiety disorders
		F41.- Other anxiety disorders
		F42.- Obsessive-compulsive disorder
		F43.- Reaction to severe stress, and adjustment disorders
		F43.0 Acute stress reaction
		F43.1 Post-traumatic stress disorder (PTSD)
		F43.2; 43.8; 43.9 Adjustment disorders
		F44.- Dissociative (conversion) disorders
		F45.- Somatoform disorders
		F48.- Other neurotic disorders
	Schizophrenia, schizotypal and delusional disorders	F20.- Schizophrenia
		F21.- Schizotypal disorder
		F22.- Persistent delusional disorders
		F23.- Acute and transient psychotic disorder
		F24.- Induced delusional disorder
		F25.- Schizoaffective disorders
		F28.- Other nonorganic psychotic disorders
		F29.- Unspecified nonorganic psychosis
Routine care data—prescriptions		Antipsychotics (N05A)
		Anxiolytics (N05B)
		Hypnotics and sedatives (N05C)
		Antidepressants (N06A)
		Psychostimulants (N06B)
		Anti-dementia drugs (N06D)
		St. John’s wort (N05CP03, N06AP01, N06AP51)
		Valerian root (N05CP01, N05CP51, N05CP06)
		Lavender oil (N05BP03)
		Ginkgo-biloba (N06DP01, N06DA53)
Routine care data—referrals		Internist/Internal Medicine (LANR 23)
		Cardiologist (LANR 28)
		Neurology (LANR 53)
		Neurology and Psychiatry (LANR 51)
		Psychiatry and Psychotherapy (LANR 58)
		Psychosomatic Medicine and Psychotherapy (LANR 60)
		Psychotherapeutic doctor (LANR 61)
		Psychological psychotherapist (LANR 68)
Routine care data—billing code	Billing for services provided by the statutory health insurance funds (“*Einheitlicher Bewertungsmaßstab*”, EBM)	01612: Consultation report before psychotherapy
		03230: Problem-oriented medical discussion necessitated by the nature and severity of the illness. Counselling and discussion of the therapeutic, family, social or professional consequences and their management in connection with the disease(s) which, due to their nature and severity, make the interview necessary, per 10 min completed
		03212: Supplement to the flat rates for insured persons according to Nos. 03110 to 03112 for the treatment of an insured person with one or more serious chronic illness(es)
		03242: Test procedure for suspected dementia
		03360: General practitioner-geriatric basic assessment
		03362: General practitioner-geriatric care complex
		30980: Clarification before the implementation of a further geriatric assessment according to the fee schedule item 30984 by a physician
	Billing for services provided by private health insurance companies (“*Gebührenordnung für Ärzte*”, GOÄ)	3: In-depth counselling that exceeds the usual level, also by telephone
		8: Full body status incl. orienting neurological examination
		15: Accompanying therapeutic and social measures for the chronically ill
		800: Detailed neurological examination
		801: Detailed psychiatric examination
		835: Taking the medical history of others
		857: Orienting test examinations (MMST, BDI)
Routine care data—organisational statistics	Workload	Number of patients treated per PCP per day

## Data Availability

Not applicable.
